# Can Individual Agency Compensate for Background Disadvantage? Predicting Tertiary Educational Attainment among Males and Females

**DOI:** 10.1007/s10964-020-01290-2

**Published:** 2020-07-24

**Authors:** Ingrid Schoon, Rose Cook

**Affiliations:** 1 University College London, Institute of Education, London, UK; 2 Kings College London, London, UK

**Keywords:** Agency, Social origin, Educational mobility, Gender

## Abstract

Adolescent agency has been identified as a central aspect in the study of social mobility and status attainment. There is however still a lack of understanding of (a) how different SES dimensions influence the expression of multiple dimensions of agency; (b) the interplay of SES and adolescent agency in shaping adult outcomes; and (c) variations in these associations by gender. Focusing on educational mobility, this study adopts a multiple exposure multiple outcome approach specifying the associations between multiple SES dimensions and multiple indicators of domain-specific agency and their relative role as predictors of educational attainment, also testing for potential gender differences. The study draws on data collected for the nationally representative Longitudinal Study of Young People in England, comprising 6719 individuals born in 1989/90 (48% female; 85% first generation students; 15% ethnic minority status). The findings show that multiple SES indicators independently influence the expression of different agency dimensions, in particular regarding educational intentions and success expectations. Moreover, multiple dimensions of education-related agency are significant predictors of enrolment in university by age 20 and degree completion by age 25, after controlling for family SES, ethnicity, and prior academic attainment. The evidence points to mainly independent agency effects and provides some support for compensatory effects regarding school engagement. Although females report higher levels of education-related agency, the manifestation of agency benefits both males and females equally. The findings suggest that critical insights into social mobility processes can be gained when using more complex models that take into account multiple dimensions of SES and agency and their interactions over time.

## Introduction

Attaining a degree level qualification is a highly valued and desired developmental outcome. However, not all young people have the same opportunities to pursue this goal. There are persisting social inequalities, and family socio-economic disadvantage is widely recognised as a key predictor of educational attainment (OECD 2018). Young people from disadvantaged families are less likely than their more privileged peers to apply to university, and ultimately to enrol in and complete tertiary education (Johnson and Reynolds 2013; Schoon and Lyons-Amos 2017), even after taking into account general levels of cognitive ability (Schoon 2010). However, not all young people from relatively disadvantaged family backgrounds fail to achieve, and some succeed against the odds. Prior research has shown that different dimensions of agency such as academic self-concept (Marsh and O’Mara 2008), educational aspirations (Schoon 2006), as well as expectations of success (Shane and Heckhausen 2017) are important predictors of educational attainment, even among disadvantaged youth. However, existing studies tend to examine different dimensions of agency and family socio-economic status (SES) in isolation (or as undifferentiated summary measures), and there is a lack of understanding regarding their relative and independent effect in shaping educational progression. Moreover, there is still little attention to potential gender differences in the processes through which SES and agency influence educational attainment. To address these limitations, the current study examines (1) the role of multiple SES dimensions in shaping adolescent agency; (2) whether distinct dimensions of adolescent agency can compensate for SES disadvantage in determining educational attainment; and (3) potential gender differences in the associations between SES, adolescent agency and educational attainment.

### Adolescent Agency

During adolescence, a number of important decisions have to be made regarding which educational pathway to take, whether to continue in higher education or to prepare for an early entry into the labour market. The capacity to make decisions and efforts to steer the course of one’s own life have been referred to by the construct of human agency (Bandura 2006; Emirbayer and Mische 1998). Individual agency is generally understood as a multi-dimensional construct. This applies within sociological (Emirbayer and Mische 1998; Hitlin and Elder 2007) and psychological (Bandura 2006; Eccles 2008) approaches. At the most general level, agency can be conceptualised as intentional action, involving an orientation to the future (e.g. expression of future goals or intentions), reflections on past behaviour and experience (e.g. self-concepts), perceptions of one’s capabilities within given constraints (e.g. expectations of success), and perceived self-regulation (e.g. following one’s own values in the selection and pursuit of one’s goals). However, previous research on the role of agency in shaping educational mobility has focused on only one or two of these dimensions, and the systematic investigation of the relative and independent contributions of different agency dimensions, and their interactions with structural factors in shaping educational progressions, is lacking.

Empirical research on agency has been hampered by differences in terminology, level of analysis and approaches to measurement used across disciplines (Hitlin and Long 2009).While sociological research emphasises processes of social reproduction, psycholocial research focuses on the conceptualisation and assessment of individual volition and the human capacity to transcend immediate constraints. Integrative approaches, drawing on both sociological and psychological assumptions, recognise that the manifestation of agency reflect a relational and dynamic process, a phenomenon that emerges through the interplay of individual capacity and the socio-cultural structures encountered in the family, the school and/or the wider social context (Johnson and Hitlin 2017; Schoon and Heckhausen 2019).

To specify the focus of empirical research, a differentiation can be made between domain-general indicators of agency, i.e. those that are related to a range of different outcomes across the life course; and domain-specific indicators of agency, i.e. indicators assessed at the same level of specificity as the outcome (Hitlin and Johnson 2015). Given the emphasis of this study on educational attainment, a range of domain-specific facets of adolescent agency are considered, which have been established as important predictors of educational attainment. These include academic intentions (Reynolds and Johnson 2011; Vaisey 2010), academic self-concepts (Marsh and O’Mara 2008; Parker et al. 2018), academic success expectations (Shane and Heckhausen 2017), and school engagement (Schoon 2008). Examining the role of multiple dimensions of agency enables us to assess whether educational mobility requires only one dimension (e.g. high educational expectations or high levels of school engagement) or multiple dimensions. The former constellation indicates a sufficient condition, while the latter reflects multiple necessary causes (Schoon and Heckhausen 2019). Studying multiple dimensions of agency simultaneously can thus help illuminate their relative and combined role in shaping educational progression.

### SES and Agency

Previous research has shown that individual agency is influenced by family socio-economic background and resources, as reflected in the term ‘bounded or structured agency’ (Evans 2002; Shanahan 2000). Young people from relatively disadvantaged backgrounds face greater difficulties when developing ambitious educational and career goals, because they tend to feel constrained by perceptions of limited opportunities and resources. Their ‘horizon of perceived possibilities’ tends to be foreshortened (Schoon and Heckhausen 2019), they express lower educational expectations (Reynolds and Johnson 2011; Vaisey 2010) and academic self-confidence than their more privileged peers (Schoon 2014).

Although the role of SES in shaping individual agency has been extensively studied, SES has been operationalised in different ways. SES is commonly referred to as a multi-dimensional construct, and there is considerable diversity in the choice of indicators used to create a valid measure (Bukodi and Goldthorpe 2013; Sirin 2005). Depending on discipline, the focus in operationalising SES in previous studies had been on parental education, income or social class. These different facets of SES relate to different forms of parental resources, including economic, socio-cultural and informational resources, which in turn have shown to have independent and distinct effects on educational attainment (Bukodi and Goldthorpe 2013; Pensiero and Schoon 2019). Inconsistency in operationalization of SES, in turn, can lead to variable findings regarding the association between SES and agency, or educational attainment outcomes for that matter (Goldthorpe 2013). Moreover, there have been significant changes regarding the association between parental socio-economic background and opportunities for upward mobility in the aftermath of the massive education expansion since the 1980s. Increasing numbers of young people from disadvantaged backgrounds aspire to go to university, reflecting a new norm of ‘college for all’ (Rosenbaum 2001), and the association between parental socio-economic status and achievement orientations has diminished (Reynolds and Johnson 2011; Schoon 2010). There is, however, still a lack of understanding of the extent to which different facets of family SES influence a range of different agency dimensions among current cohorts of young people.

Moving beyond a unidimensional conceptualisation of SES and agency, a multiple-exposure multiple outcome (MEMO) approach is adopted in this study, assessing the relative and independent influence of different SES dimensions on the manifestation of multiple dimensions of adolescent agency. Such a multi-dimensional approach to the study of exposure to adversity and associated multiple outcomes is promoted in resilience research (Schoon 2006), acknowledging that different dimensions of adversity tend to cluster together (Schoon and Melis 2019), and that it is generally not one component, but their combined effect that matters. Moreover, the impact of adversity is not the same for different outcomes, and unless multiple outcomes are considered, only a partial picture of risk exposure can be formulated.

In assessing the interactions between SES and agency in shaping educational transitions, the current study interrogates three distinct hypothetical processes identified in previous studies. First, according to the *independent effect hypothesis* (no interaction), it can be assumed that agency indicators predict transition outcomes independent of structural constraints, i.e. both structure and agency have a unique contribution to transition experiences and agency is not simply a proxy for SES. For example, previous research has shown that achievement goals, such as expectations to participate in higher education or to enter a professional career, are associated with subsequent educational and occupational attainment, over and above the influence of social background and cognitive ability (Johnson and Reynolds 2013; Schoon 2010a, 2010b), as are subjective expectations of success (Ashby and Schoon 2010) and indicators of self-regulation (Moffitt et al. 2011; Ng-Knight and Schoon 2017). Second, previous research also suggests *cumulative effects*, in that different resources multiply each others’ impact (Ross and Mirowsky 2006). An advantaged social position is associated with resources critical for further relative gains, such as high educational expectations as well as access to financial, social and cultural support, while a disadvantaged position is generally associated with a lack of resources. Thus, (dis)advantages tend to accumulate or multiply over the life course (Dannefer 2003), and young people from less privileged background tend to express lower levels of agency than their more privileged peers, which in turn, is associated with lower educational attainment (Reynolds and Johnson 2011; Vaisey 2010). It can thus be assumed that agency is more effective for already privileged young people. Third, according to a *compensatory effect hypothesis* it is assumed that agency may be more important for young people with fewer socio-economic resources. Potential ‘compensatory effects’, also described by the term ‘resource substitution’ (Ross and Mirowsky 2006), refer to processes where one resource can substitute for another or can fill the gap if the other is absent. For example, adolescents from a less privileged family may depend more on their individual level agency to succeed in education than their more privileged peers. There is some evidence to that effect in previous studies (Hitlin and Johnson 2015; Ng-Knight and Schoon 2017; Schoon and Lyons-Amos 2017), although there is still a lack of understanding if compensatory or cumulative effects apply to both males and females.

### Gender Differences in Agency and Educational Attainment

Females generally have higher educational aspirations and are also more likely to participate in higher education than males (Berrington et al. 2016; Schoon and Eccles 2014). From early childhood, females outperform males on many of the ‘non-cognitive’ traits that are associated with long-term academic attainment. For example, girls score higher than boys on scales of social and behavioural skills and ‘approaches to learning’ in early childhood, such as attentiveness, task persistence, eagerness to learn, learning independence, flexibility and organisation (DiPrete and Jennings 2012). Girls are rated by their teachers as more engaged and attentive at school (Downey 2005; Lietaert et al. 2015). Academic self-concept is the exception: males tend to have higher academic self-concept, conditional on attainment (Parker et al. 2018). Yet, females have generally higher education expectations than males (Berrington et al. 2016; Schoon and Eccles 2014), also among youth from less privileged background (Schoon 2010).

Meanwhile, males are also at greater risk of not progressing to higher education when they come from more disadvantaged origins. Buchmann and DiPrete (2006) highlight the growing vulnerability of males to absent or less educated fathers; the female advantage in higher education emerged first in such families. Subsequent research confirms that socio-economic disadvantage has a disproportionate impact on males in terms of educational outcomes (OECD 2015). In contrast, females are less influenced by their parents and family background when deciding whether to continue to higher education (Schoon 2010).

Taken together, the evidence suggests that females from diverse socio-economic backgrounds tend to have more agentic resources than males to support key educational transitions, such as enroling in and completing university. However, though it seems that males are more susceptible to the impact of socio-economic disadvantage, the intersection of gender, agency and family SES has rarely been studied. Since most of the agency characteristics that support educational transitions are more common in females, regardless of socio-economic background, and scarcer among males, they may confer a greater advantage when present among males. Males with high expectations, self-concept, expectations of success and school engagement may stand out from their peers and attract support from teachers and parents in their academic endeavours. In contrast, among females, where these resources are more common, they may confer less of a comparative advantage. It could thus be the case that there are stronger associations between agency and attainment for males compared to females. It could however also be the case, following the assumption of cumulative resources, that agency provides more of an advantage for females. As already mentioned, the exception to this is academic self-concept, which is expected to be higher among males and thus to have more of an impact for females given its scarcity among females, or vice versa. As there is little empirical evidence on potential gendered experiences, both assumptions are explored.

## Current Study

Previous research has shown that different dimensions of agency are important predictors of educational attainment, even among disadvantaged youth. However, most prior studies focused on distinct dimensions of agency separately or used undifferentiated summary measures, and it is unclear whether different dimensions of agency have independent or combined roles in shaping educational transitions. Moreover, most studies did not conceptualise SES in multi-dimensional terms, in spite of evidence that factors such as parents’ occupational background, parents’ education, and income, have unique and independent impacts on educational outcomes. In addition, the intersection of SES, agency and gender in shaping educational attainment has received little attention in previous research. Drawing on data from a nationally representative longitudinal study and adopting a MEMO approach, the current study asks how do different dimensions of adolescent agency and their effect on future educational attainment vary by family SES and gender. First, the associations of multiple dimensions of agency and different SES components are examined. Second, the interplay of multiple dimensions of family SES and adolescent agency in shaping adult educational attainment is scrutinised, assessing their relative and independent influence, and testing potential cumulative versus compensatory processes. Third, as there is little empirical evidence on the gendered role of agency in predicting educational mobility, the question whether agency has a stronger role for male or for females is explored.

Given that drop-out rates in higher education are a persistent concern, and non-completion rates in the UK are above 35% (OECD 2009), the study examines the associations between indicators of adolescent agency assessed at age 13/14 (in 2004), enrolment at university by age 20 (in 2010) and completion of a degree-level qualification by age 25 (in 2015)—moving beyond approaches that focus on educational attainment only to consider educational progression. Moreover, this study controls for prior academic attainment and ethnic minority status to take into account potential confounding factors. In the UK young people from ethnic minority background generally express higher education aspirations than their male white peers and are also more likely to participate in higher education (Berrington et al. 2016; Strand 2014).

## Methods

### Data

The study is based on data collected for Next Steps (formerly known as the Longitudinal Study of Young People in England (LSYPE)). Next Steps is a panel study of young people born between 1st September 1989 and 31st August 1990. Sample members were all young people in school year 9 (age 13/14) or equivalent, in England in February 2004. Schools in deprived areas were oversampled, as were ethnic minorities, and special design weights are used in the analysis (for more details see (Department for Education 2011). Annual face-to-face interviews have been conducted with young people and their parents between 2004 and 2010, and linkage is available to other administrative data, such as the National Pupil Database (NPD), which includes national assessments for all children in England. In 2013 responsibility for the study shifted from the UK Department of Education to the Centre for Longitudinal Studies (CLS) which restarted the study under the name Next Steps and conducted a follow-up study in 2015 when the study members were aged 25, comprising a sample of 7707 respondents (Calderwood 2018).

### Analytic Sample

As all longitudinal studies, Next Steps experienced sample loss between the multiple waves. The sample used for this study comprises respondents to the age 25 survey (*n* = 7707). The analytic sample includes 6719 individuals (3046 males and 3673 females) with complete data on gender, ethnicity, and the auxiliary variables used in the multiple imputation to address issues of missing data (see section on modelling strategy). The sample is largely representative of the original sample, although drop out of the survey by Wave 8 was predicted by parents being unmarried, having low education, being unemployed, being in a lower occupational group, and ethnic minorities. Drop out was also predicted by the agency variables central to the study. Special sample weights have been calculated to account for selective attrition from the survey (Calderwood 2018) and these are used in all analyses.

### Measures

#### Agency

Four different facets of domain-specific agency were assessed at wave 1 (age 13/14).

***Academic expectations (intention)****.* The young people were asked how likely it is that they will ever apply to go to university to do a degree. Responses were coded on a 4-point scale with response options 4 = Very likely, 3 = Fairly likely, 2 = Not very likely, and 1 = Not at all likely.

***Success expectations***. Young people who indicated that they would apply to university were asked how likely they think it is that if they do apply that they will get in. Responses were coded on a 4-point scale with response options 4 = Very likely, 3 = Fairly likely, 2 = Not very likely, and 1 = Not at all likely. Those who are filtered out (i.e. those who think it is ‘not at all likely’ they will apply to HE based on previous question and are not asked this question) are given a value of 0 in the models (so that 0 = don’t know/not applicable; 1 = not at all likely; 2 = not very likely; 3 = fairly likely; 4 = very likely).

***Academic ability concepts***. Perceived efficacy to master different academic subjects was measured by asking the young people how good they would say they are in math, English, science and Information and Communications Technology (ICT). Responses were coded on a 4-point scale with response options 4 = Very good, 3 = Fairly good, 2 = Not very good, and 1 = Not good at all. The items were summed up to create an index of academic self-efficacy (alpha = 0.53). The scale score was z-standardised. A high score indicates high and a low score low levels of academic ability concepts.

***School engagement****.* A scale score was created based on summed answers to 5 attitudinal statements. Statements include: I am happy at school; school work is worth doing; I work as hard as I can at school; I am bored in lessons; on the whole I like being at school. Responses were coded on a 4-point scale with response options 4 = Strongly disagree, 3 = Disagree, 2 = Agree, 1 = Strongly agree. The scale has good internal consistency (alpha = 0.73). The scale score was z-standardised, and a high score indicates positive school engagement and a low score school disengagement.

#### Educational attainment

Educational progression was assessed with two indicators: enrolment at university by age 20 and completion of a degree level qualification by age 25.

#### Family socio-economic resources (SES)

Four dimensions of family socio-economic resources were assessed at wave 1.

***Parental education*** differentiates parents with degree level qualifications and all levels below (including qualifications at level 3 which enable access to higher education; qualifications up to level 2 (the General Certificate of Secondary Education (GCSE); and no qualifications).

***Parental social class*** is assessed using the National Statistics Socio-Economic Classification (NS-SEC), differentiating between higher managerial/professional, intermediate, routine/manual occupations and the long-term unemployed. Following the dominance approach (Erikson, 1984) the highest education level and social class of either parent was identified.

***Gross household income*** was reported by the main parent and coded into quintiles.

***Home ownership*** in Wave 1 is coded as 1 if the family owns their own home and 0 if they are renting.

To simplify interpretation of interaction terms, a socio-economic risk index was created using Principal Components Analysis of the four SES indicators (parental education, NS-SEC occupation, family income level and home ownership) where higher scores indicate lower levels of socio-economic resources. The Principal Component Analysis suggests one underlying dimension that explains 69% of the variance in the four SES indicators, with average inter-item covariance of 0.45.

#### Control variables

The analyses controlled for the adolescents’ gender (0 = Male) (1 = Female), ethnicity, and prior academic attainment. Ethnicity was coded as (0) White, versus (1) Other ethnic groups. Given the ethnic diversity in England, the different ethnic groups were too numerous and the number of each group was too small to examine differences among the groups individually. Academic performance at age 11 was measured using information from the National Pupil Data (NPD), creating a latent variable comprising math, English and science scores in national curriculum tests (Key Stage 2 or KS2) given by age 11 (years 3–6).

#### Modelling strategy

Ordinal logistic regression models and OLS regression models were estimated to test the associations between dimensions of family SES and agency variables. Models were run in a stepwise fashion, first adding only the SES indicators (Model 1) before adding controls for prior attainment, gender and ethnicity (Model 2). Subsequently, the role of agency variables and family SES in shaping educational attainment was tested for males and females separately using logistic regression models, focusing on two outcomes: (1) enroled in higher education at age 20 and (2) completed a degree at age 25. These models were run in two stages with Model 1 including agency variables only and Model 2 adding socio-economic background and control variables.

Sample weights provided with the survey account for attrition or drop out from the survey over successive waves, which is quite significant in the case of Next Steps (Calderwood 2018). However, weights do not account for missing data on individual variables. To retain adequate sample sizes and avoid losing information on young people with missing data, multiple imputation was used for independent and dependent variables. Analysis was conducted using the MI command in STATA (Royston 2004), creating 20 data sets. Auxiliary variables used in the imputation model were carefully selected to be highly correlated with variables to be imputed and to have a low level of missingness themselves: these were area-level deprivation, and educational achievements tests given by age 14 (key stage 3) and by age 16 (key stage 4). Missingness on the variables included in the model varied from 0 to 23%, with the highest level of missing data on information regarding household income. All analyses were carried out using the software package STATA15.

## Results

Table 1 shows descriptive statistics for all variables for the whole sample, split by gender. About 35% of the sample enroled in higher education, which reflects the national enrolment rate of 37% among 17–20 year olds at the time (Department for Education 2018). More young women than young men enrol in and complete university. At age 14, the majority of students (64%) say they are likely to apply to university, although a considerable number don’t. Girls were more likely to say they would apply to university and had higher expectations of success, as well as higher school engagement. Girls also have slightly higher age 11 educational attainment, suggesting that the agentic and attainment resources to succeed in educational transitions are higher among girls than boys. Contrary to expectations, no significant gender differences in academic self-concept were found. There were also no gender differences in family socio-economic resources.

**Table 1:** Descriptive statistics

	All (*N* = 6719)	Females (*N* = 3673)	Males (*N* = 3046)
Outcome variables			
Enroled in university at age 20 (%)	34.80%	**38.44%**	31.39%
Degree by age 25 (%)	25.40%	**27.50%**	23.52%
Agency variables (age 13/14)			
Likely to apply to HE (mean 1–4 scale)	2.76	**2.86**	2.67
Not at all likely (%)	16.90%	13.12%	20.50%
Not likely (%)	19.40%	19.85%	18.93%
Fairly likely (%)	34.30%	35.02%	33.74%
Very likely (%)	29.30%	32.01%	26.83%
Expectations of success (mean 1–4 scale)	3.07	**3.22**	2.94
Not applicable (%)	17.90%	13.95%	21.57%
Not at all likely (%)	1%	1.37%	1.09%
Not likely (%)	12.80%	12.78%	12.87%
Don’t know (%)	5.90%	6.88%	4.98%
Fairly likely (%)	48.10%	50.72%	45.69%
Very likely (%)	14.00%	14.30%	13.80%
School engagement (mean Z-score)	−0.13	**−0.10**	−0.17
Academic self-concept (mean Z-score)	−0.08	−0.06	−0.10
Family socio-economic variables (Wave 1)			
Parents’ education			
Degree (%)	14.90%	14.66%	15.03%
Below degree (%)	85.10%	85.34%	84.97%
Parents’ NS-SEC			
Professional and Managerial (%)	34.10%	35.54%	32.74%
Intermediate (%)	32.20%	31.75%	32.79%
Routine & Semi-routine (%)	28.20%	27.12%	28.99%
Long-term unemployed (%)	5.50%	5.59%	5.48%
Household income			
Quartile 1 (highest) (%)	21.00%	21.25%	20.83%
Quartile 2 (%)	19.00%	19.13%	19.04%
Quartile 3 (%)	25.40%	24.22%	26.28%
Quartile 4 (%)	16.10%	17.13%	15.32%
Quartile 5 (lowest) (%)	18.50%	18.27%	18.53%
Home ownership (%)	31.40%	31.07%	31.70%
Control variables			
KS2 achievement (mean points)	26.83	**27.03**	26.65
Female (%)	48.10%		
Ethnic minority (%)	14.60%	14.64%	14.55%

Significant gender differences are given in bold; KS2 refers to academic performance at age 11

Table 2 shows that young people whose parents have themselves obtained a university degree are more likely to enrol in and complete university, compared to young people with less educated parents, and that women are more likely to engage in higher education than men. Overall, the proportion of women enroled in university by age 20 is 38.4% compared to 31.4% of males. Higher education participation among women with degree-educated parents is 70.1% and among men with degree-educated parents it is 65.4% compared to 33.8% of females and 25.6% of males with parents who are educated below degree level.

**Table 2:** Identification of educational mobility (educational attainment by parental education)

	At Uni by age 20 (34.8%)		Degree by age 25 (25.8%)	
Parental education	Male	Female	Male	Female
Degree level (14.9% of parents)	65.39%	70.08%	51.13%	49.96%
Below degree level	25.65%	33.77%	18.93%	24.02%
All	31.39%	38.44%	23.52%	27.50%
N	3046	3673	3046	3673

The proportions of young people completing a degree by age 25 are considerably lower than the age 20 enrolment rates, suggesting a substantial level of dropout. For example, among young women, 27.5% have completed a degree by 25, compared to the nearly 40% who enroled. The level of dropout is particularly high among those with degree-educated parents, for both males and females. Though the overall levels of degree completion are lower among young people with low-educated parents, the difference between completion rates and initial enrolment rates is considerably less. For example, among young men whose parents are educated below degree level, 25.6% enrol and 18.9% complete a degree by 25. In contrast, 65.4% of young men with degree-educated parents initially enrol in university, while only 51.1% completed a degree by age 25, suggesting that nearly a third of those originally enroled do not complete their degree. This implies that young people from less educated backgrounds may be more persistent in pursuing their educational goals, perhaps supported by agency.

### Associations between SES and Agency

Table 3a, b show the association between the socio-economic background variables and the four agency indicators, using a stepwise modelling approach. Table 3a shows the associations for young people’s likelihood of applying to higher education, and their expectations of success using ordinal logit regression. Table 3b shows the associations for school engagement and academic self-concept, using OLS regressions. In both tables, Model 1 includes the SES background variables, while Model 2 controls additionally for gender, ethnicity and prior academic attainment.

**Table 3:** a. Association between SES indicators and domain-specific agency variables (intention to apply to HE and expectations of success): ordinal logistic regression models (odds ratios)

	Likely to apply to HE				Expectations of success			
	Model 1		Model 2		Model 1		Model 2	
	OR	SE	OR	SE	OR	SE	OR	SE
Parents’ education (ref: degree)								
Below degree	0.44***	0.04	0.55***	0.05	0.48***	0.04	0.65***	0.06
NSSEC (ref: Professional/managerial)								
Intermediate	0.66***	0.05	0.73***	0.05	0.70***	0.05	0.79***	0.06
Routine/semi routine	0.53***	0.06	0.66***	0.06	0.56***	0.05	0.71***	0.06
Long term unemployed	0.67*	0.11	0.74	0.13	0.60**	0.11	0.71	0.13
Income (ref: Q1)								
Q2	0.81*	0.07	0.88	0.08	0.78**	0.07	0.86	0.08
Q3	0.77**	0.08	0.83*	0.07	0.82*	0.07	0.90	0.08
Q4	0.72**	0.08	0.73*	0.09	0.75*	0.09	0.80*	0.09
Q5	0.70**	0.09	0.75*	0.10	0.71*	0.09	0.78*	0.09
Home ownership (ref: own home)								
Renting	0.89	0.07	1.13	0.09	0.87	0.07	1.10	0.09
Controls								
Female			1.34***	0.08			1.20**	0.07
(ref: male)								
KS2 achievement			1.22***	0.01			1.22***	0.01
Ethnic minority (ref: white)			4.26***	0.37			3.63***	0.33
Cut 1	−3.21	0.10	2.59	0.29	−2.42	0.09	3.59	0.30
Cut 2	−2.03	0.09	3.93	0.30	−2.35	0.09	3.67	0.30
Cut 3	−0.42	0.08	5.80	0.30	−1.72	0.09	4.40	0.30
Cut 4					0.72	0.09	7.17	0.32
N = 6719								

b. Association between SES indicators and domain-specific agency variables (School Engagement and Academic Self-Concept): OLS regression models.								

	School engagement				Academic self-concept			
	Model 1		Model 2		Model 1		Model 2	
	Coef.	SE	Coef.	SE	Coef.	SE	Coef.	SE
Parents’ education (ref: degree)								
Below degree	−0.14***	0.04	−0.07	0.04	−0.18***	0.04	−0.10**	0.04
NSSEC (ref: Professional/managerial)								
Intermediate	−0.02	0.04	0.01	0.04	−0.08*	0.04	−0.05	0.04
Routine/semi routine	−0.11	0.06	−0.05	0.05	−0.15**	0.05	−0.08	0.05
Long term unemployed	−0.06	0.10	−0.08	0.10	−0.05	0.10	−0.04	0.10
Income (ref: Q1)								
Q2	−0.10	0.05	−0.08	0.05	−0.10	0.05	−0.08	0.05
Q3	−0.11*	0.05	−0.10	0.05	−0.13**	0.05	−0.11*	0.05
Q4	−0.08	0.07	−0.08	0.07	−0.13*	0.07	−0.12	0.06
Q5	−0.13	0.07	−0.12	0.07	−0.18**	0.07	−0.16*	0.07
Home ownership (ref: own home)								
Renting	−0.17***	0.04	−0.13	0.04	−0.11***	0.04	−0.06	0.04
Controls								
Female			0.07*	0.03			0.05	0.03
(ref: male)								
KS2 achievement			0.04***	0.01			0.04***	0.01
Ethnic minority (ref: white)			0.40***	0.04			0.36***	0.04
Cons	0.16	0.04	−1.01	0.17	0.29	0.04	−0.99	0.16
N = 6719								

Model 1 includes SES indicators only. Model 2 controls additionally for gender, academic performance at age 11 (KS2) and ethnicity

*p < 0.05, **p < 0.01, ^***^p < 0.001

Young people’s intentions to apply to university and their expectations of success are shaped by family socio−economic background (Table 3a). Moreover, the different dimensions of socio-economic resources show independent effects on the various aspects of agency. For example, the log odds of applying to higher education for young people with parents educated below degree level are around half that of young people with degree-educated parents, after taking into account all other SES variables. These associations reduce but remain significant when controlling for gender, ethnicity and prior academic attainment. Parental education is also associated with expectations of success, school engagement and academic self-concept (Table 3b), though the association is no longer significant for school engagement once the additional controls are added in Model 2. Parental social class also affects young people’s higher education intentions and expectations of success. However, there is no association with school engagement and associations with academic self-concept are small and disappear in Model 2. Being in a lower household income group is associated with lower intentions to apply to university, lower expectations of success and lower academic self-concept, even when controlling for prior attainment—but income is not strongly associated with school engagement. Home ownership is associated with young people’s school engagement and academic self-concept, but not with the other agency variables, and associations do not hold once controls are added. In terms of the control variables, girls have higher intentions to apply to university, higher expectations of success and higher school engagement. Compared to their white British peers, young people from ethnic minority backgrounds have higher scores on all agency variables. All agency variables are positively associated with higher levels of prior academic attainment.

The findings suggest that social background shapes agency, but the associations vary across components of socio-economic background and agency. In particular, socio-economic background exerts a more consistent influence on higher education intentions and expectations than on school engagement and academic self-concept. Especially parental education and also social class appear to matter more regarding their influence on educational intentions and success expectations than household income or home ownership.

### Independent Effects of Agency and SES in Shaping Educational Attainment

In a first step to assess the influence of agency on educational attainment, the *independent effect hypothesis* was tested for the outcomes being enroled in university at age 20 (Table 4) and completing a degree by 25 (Table 5). A stepwise logistic regression approach was used. Model 1 only includes the individual agency variables as predictors. Model 2 adds a full set of control variables to see whether any associations observed in Model 1 still hold. The ‘independent effect’ models are run first for the whole sample of young people and subsequently separately for males and females, to see if there are any gender differences in the way that agency variables are associated with educational transitions. The statistical significance of these gender differences was further assessed using interactions between gender and each agency variable in pooled models.

**Table 4:** Association between Individual agency and being enroled in higher education at age 20: logistic regression models (odds ratios)

	All		Males		Females	
	M1	M2	M1	M2	M1	M2
Agency						
Likely to apply to HE	1.91***	1.49***	1.83***	1.48***	1.96***	1.49***
Expectations of success	1.26***	1.09	1.31***	1.1	1.21***	1.07
School engagement	1.43***	1.50***	1.43***	1.56***	1.43***	1.47***
Academic self-concept	1.39***	1.31***	1.43***	1.34***	1.35***	1.28***
Parents’ education (ref: degree)						
Below degree		0.54***		0.53***		0.57***
Parents’ NSSEC (ref: Professional/managerial)						
Intermediate		0.75**		0.66**		0.84
Routine/semi routine		0.62***		0.57**		0.68*
Long term unemployed		0.66		0.68		0.64
Household income						
(ref: Q1)						
Q2		0.76*		0.71		0.80
Q3		0.72**		0.73		0.71*
Q4		0.76		0.91		0.65*
Q5		0.79		0.8		0.77
Home ownership (ref: own home)						
Renting		0.55***		0.54***		0.55***
Controls						
Female (ref: male)		1.32***				
KS2 achievement		1.28***		1.29***		1.27***
Ethnic minority (ref: white)		3.19***		3.13***		3.29***
Constant	0.01	0.00	0.03	0.00	0.04	0.00
N	6719	6719	3046	3046	3673	3673

KS2 refers to academic performance at age 11

^*^p < 0.05, ^**^p < 0.01, ^***^p < 0.001

**Table 5:** Association between individual agency and having a degree by age 25: logistic regression models (odds ratios)

	All		Males		Females	
	M1	M2	M1	M2	M1	M2
Agency						
Likely to apply to HE	1.78***	1.42***	1.78***	1.47***	1.76***	1.39***
Expectations of success	1.19***	1.04	1.19**	1.1	1.18***	1.06
School engagement	1.25***	1.26***	1.31***	1.56***	1.20**	1.18*
Academic self-concept	1.25***	1.17**	1.29***	1.34***	1.21**	1.15*
Parents’ education (ref: degree)						
Below degree		0.62***		0.53***		0.71**
NSSEC (ref: Professional/managerial)						
Intermediate		0.91		0.66**		0.93
Routine/semi routine		0.72**		0.57**		0.67**
Long term unemployed		0.68		0.68		0.72
Income (ref: Q1)						
Q2		0.79*		0.71		0.92
Q3		0.79*		0.73		0.94
Q4		0.88		0.91		0.85
Q5		0.84		0.8		0.82
Home ownership (ref: own home)						
Renting		0.71***		0.54***		0.71*
Controls						
Female (ref: male)		1.13				
KS2 achievement		1.21***		1.29***		1.19***
Ethnic minority (ref: white)		2.29***		3.12***		2.46***
Constant	0.03	0.00	0.03	0.00	0.04	0.00
N	6719	6719	3046	3046	3673	3673

KS2 refers to academic performance at age 11

^*^p < 0.05, ^**^p < 0.01, ^***^p < 0.001

Table 4 shows that each of the agency variables are independently associated with the likelihood of being enroled in higher education at age 20. The intention to apply to HE at age 14 shows the strongest association with enrolment for both males and females (OR: 1.91; males 1.83; females 1.96). School engagement and academic self-concept have smaller but significant associations with higher education enrolment for both males and females. When controlling for the full set of control variables (Model 2) all agency indicators remain significant predictors, except for expectations for success. Interestingly, the role of school engagement appears to be stronger when control variables are included, indicating potential suppressor effects.

Table 5 shows the independent agency effects with “completion of a degree at age 25” as the outcome. All agency variables measured at age 14 show significant associations with completing a degree by age 25. After taking into account the control variables (Model 2) these associations reduce but remain significant apart from expectations of success, mirroring the results in Table 4. Among males, the role of school engagement and academic self-concept appears slightly stronger when adding the control variables.

Overall, these results confirm the *independent effect hypothesis*: age 14 higher education intentions, school engagement and academic self-concept have a long-run effect on educational transitions, over and above the effect of socio-economic background and academic attainment. The role of success expectations is less clear, since in all models it becomes non-significant once control variables are added. The influence of agency is more or less similar for males and females, providing little support for the expectation that agency plays a gendered role in predicting educational transitions. Although girls have higher levels of agency (see Table 1), agency has a similar association with educational outcomes for females and males.

### Agency: Compensatory or Cumulative Effects?

Next, the *compensatory* versus *cumulative effects* hypotheses were tested, using interaction terms to examine whether the effect of the agency variables on educational attainment is different depending on a young person’s socio-economic background. Indicators of agency were interacted with a combined indicator of the multiple SES risk dimensions (calculated from a principal component of NS-SEC social class, parental education, level of family income and housing tenure). Higher scores on this SES risk variable indicate lower levels of family socio-economic resources.

Table 6 shows significant main effects and one positive, significant interaction between SES risk and school engagement in the model predicting higher education enrolment at age 20 (*p* value: 0.02). A high level of SES risk in combination with high levels of school engagement at age 14 is associated with a higher likelihood of being enroled in university at age 20, in particular among females. The positive interaction provides some limited evidence for the *compensatory effect* or *resource substitution hypothesis* (Ross and Mirowsky 2006) assuming that agency is particularly beneficial for young people from less privileged backgrounds, enabling them to succeed against the odds.

**Table 6:** Interactions between agency variables and ses risk score: logistic regression models (odds ratios)

	Enroled at Uni at age 20			Degree by age 25		
	All	Males	Females	All	Males	Females
Agency						
Likely to apply to HE	1.56***	1.55**	1.55***	1.54***	1.54**	1.54***
Expectations of success	1.09***	1.11***	1.09	0.99	1.01	0.98
School engagement	1.57***	1.59***	1.54***	1.28***	1.39***	1.19*
Academic self-concept	1.30**	1.33**	1.28**	1.18**	1.23*	1.16*
SES risk score	0.71**	0.71	0.73*	0.74***	0.72*	0.75*
Interactions						
SES*Likely to apply to HE	0.97	0.95	0.98	1.01	1	1.01
SES* Expectations of success	1.02	1.05	1.00	1.00	1.02	0.99
SES* School engagement	1.109*	1.05	1.14*	1.06	1.06	1.07
SES*Academic self-concept	0.98	0.99	0.98	1.01	1.05	0.99
Controls						
Female (ref: male)	1.34***			1.13*		
KS2 achievement	1.28***	1.28***	1.27***	1.21***	1.23***	1.19***
Ethnic minority (ref: white)	3.46***	3.41***	3.49***	2.38***	2.26***	2.52***
Constant	0.00	0.00	0.00	0.00	0.00	0.00
N	6719	3046	3673	6719	3046	3673

KS2 refers to academic performance at age 11

^*^p < 0.05, ^**^p < 0.01, ^***^p < 0.001

### Robustness checks

All models were run using complete cases only as a comparison to the imputed results, with similar results. The models were run with parental education coded as a dichotomous and categorical variable, with similar results. The models related to Table 3a, b were also run as linear probability models, which generated similar results. Gender differences were further tested using interaction terms in a pooled model, again with similar results. Analysis for the ‘degree at age 25’ outcome models (Table 5) were run on a restricted sample of only young people who were enroled in higher education at age 20. These models highlighted the importance of higher education intentions expressed at age 14, as well as parental education and prior attainment. The other agency and socio-economic background variables were not significant in this model. The models testing for interaction terms (Table 6) were re-run with each main effect and interaction term included separately, with the same results.

## Discussion

Previous research has addressed SES influences on individual agency and subsequent educational attainment. However, most of the research focuses on distinct indicators of SES and agency in isolation ore used undifferentiated summary measures, and a more detailed understanding of the relative and simultaneous influence of different dimension of agency and SES in educational mobility processes is lacking. Analysis of gender differences in these processes are also largely absent from the literature. Moving beyond a unidimensional conceptualisation of agency and SES influences and focusing on domain-specific indicators of adolescent agency, a MEMO approach was adopted, demonstrating the relative and independent effect of different SES dimensions in shaping multiple agency indicators and examining the combined influence of SES and agency in shaping subsequent higher education enrolment and completion. Gender differences were taken into account as well as variations by ethnic minority status and prior academic attainment.

In line with previous evidence (Evans 2002; Shanahan 2000), findings show that adolescent agency is influenced by family SES. However, not all SES indicators had the same impact on the different agency dimensions. Parental education and occupation show greater effects than household income or home ownership. The most consistent influence was observed for parental education which significantly affected all four agency dimensions considered here, i.e. educational intentions, success expectations, academic self-concept and school engagement. The findings suggest domain-specific socialisation processes, with parental education being a central aspect in educational mobility processes. Other SES indicators are also relevant, in particular regarding the expression of future oriented indicators of agency, i.e. educational intentions and success expectations, but their effect is less consistent in relation to academic self-concept and school engagement. Generally, the findings highlight the importance of the MEMO approach for a better understanding of social mobility processes. Conceptualising both SES and agency as multi-dimensional constructs enables a better understanding of the underlying processes linking family socio-economic resources to the formation of achievement motivation and planning behaviours.

Regarding the interactions of SES and agency in predicting educational attainment, the findings suggest support for the *independent effect hypothesis:* different dimensions of adolescent agency show meaningful and significant main effects, over and above the influence of family SES, ethnicity and prior academic attainment. Indeed, the associations between agency and educational attainment remain mostly unchanged after adding these variables to the model, except in the case of expectations of success. Notably, multiple dimensions of agency are simultaneous predictors of educational attainment, suggesting multiple necessary conditions (Schoon and Heckhausen 2019) for educational attainment. The findings emphasise in particular the significant role educational intentions as well as school engagement (an indicator of self-regulation) and academic self-concept as unique and independent resources facilitating educational participation and completion. The influence of expectations of success on educational attainment where no longer significant once family SES, ethnic and prior academic attainment were controlled for. Maybe success expectations, i.e. certainty to be accepted if one applies to university, are more of a reflection of perceived social status advantages than the other agency indicators considered here. Regarding gender differences, the findings show that males generally expressed lower levels of education-related agency than females, reflecting the findings of previous research (DiPrete and Jennings 2012; Schoon and Eccles 2014). However, agency benefited both males and females equally in their educational progression and attainment.

The findings also suggest slight evidence in support of the *compensatory effect hypothesis* (Ross and Mirowsky 2006) in the case of school engagement and higher education enrolment. That is, school engagement at age 13/14 generally promotes higher education enrolment at age 20, yet the effect is tendentially stronger for young people (in particular females) from relatively less privileged backgrounds. There was no evidence that this compensatory effect applies for the other agency variables. Neither did the findings support the cumulative effect hypothesis, i.e. the assumption that agency is more effective among relative advantaged students. Generally, the influence of adolescent education-related agency is stronger for university enrolment than for degree completion. The findings suggest that the education-specific indicators of agency assessed here might be more effective in the school context, directing efforts towards the goal of enroling in university. However, once students have entered higher education, the academic setting might require additional competences and resources, or more general (less education focused) facets of agency to enable youth to succeed.

In interpreting the findings, the limitations of the current study must be considered. The study was bound by the data available. We only considered domain-specific agency indicators, and most of the agency variables are based on single items, which are less stable than multi-item scales. However, single-item assessments of education expectations are widely used in large scale surveys, suggesting satisfactory face validity (Vaisey 2010). In addition, the internal validity of the academic self-concept index is very low, highlighting that verbal and math self-concepts are different and the need to differentiate their distinct roles in shaping educational progression (Marsh and O’Mara 2008). The analyses controlled for prior academic attainment, since direct measures of cognitive skills are not available in the LSYPE dataset. Moreover, as in all longitudinal studies, the problem of missingness and attrition had to be tackled. Multiple imputations was used to address this issue. The robustness of findings using imputation was checked against results with complete data. Furthermore, the study reports associations between the indicators used here and cannot claim causality.

The findings might be unique to the particular age group, age cohort, and English context. The study followed a current cohort of young people born in 1989/90 up to the age of 25. Their experiences in the education system are different from earlier born cohorts, given the massive expansion of higher education since the 1980s. In addition, the study is based on young people in England, which (like the US) is characterised by a less structured education system than German speaking countries, for example (Schoon and Bynner 2019). It has been argued that the role of agency might be more important in countries will a less structured education system (Buchmann and Park 2009; Heckhausen and Chang 2009). To assess generalisability of findings it is thus necessary to compare evidence with data collected in countries with a more structured education system, such as Germany or Switzerland.

The findings show that associations between family SES and agency as well as between agency and educational attainment were only of small to moderate size. This possibly reflects the effects of increasing education expansion and a new norm of “college for all” (Rosenbaum 2001), yet leaves room for alternative explanations of persisting inequalities in educational attainment. These might include variations in the necessary financial support to pay for study fees and subsistence; knowledge about how to navigate the complex landscape of higher education or social networks facilitating getting in and getting on in higher education; differences in school characteristics and school funding; the provision of adequate and stimulating learning materials; school climate or micro-climate in the classroom; mentoring and support from teachers, or peer influences, that also play a critical role in shaping educational agency and attainment.

Moreover, the indicators of agency used in this study were assessed at only one time point. Future analysis should examine changes in expression of agency over time, and possible variation in this change by social background. Additionally, for various reasons some young people, in particular those from less privileged backgrounds, might enrol and complete higher education at a later age, given competing demands on their time and resources. Future research should thus examine in more detail the processes linking SES and agency to later educational attainment, paying special attention to the factors enabling privileged young people to achieve (such as financial and social support) versus factors that support those from less privileged backgrounds. Moreover, more attention should be paid to the intersection of SES, gender and ethnic minority status, as ethnic minorities in England are more ambitious, confident and engaged in education (Strand 2014), and are more likely to graduate than whites with similar economic and academic resources.

## Conclusion

There is a need for a more comprehensive understanding of how the expression of adolescent agency varies by family background and gender, and regarding the interactions between SES, agency and gender in shaping educational attainment. Advancing on previous research, this study conceptualises both adolescent agency and family SES as multi-dimensional constructs, enabling the assessment of their relative and independent impact on educational progression and attainment. The findings suggest processes of domain-specific socialisation, with parental education playing a central role in shaping the expression of multiple indicators of education-related adolescent agency. Different dimensions of agency, in turn, were significantly associated with later educational attainment, over and above the influence of family SES, ethnicity and prior academic attainment. The findings indicate that the independent main effect model is most appropriate for understanding the relationship between agency and participation in and completion of higher education. However, critical insights can be gained when using more complex models that also take into account interactions between SES, gender and agency. The results suggest multiple necessary conditions for young people to succeed in higher education. In particular, high levels of academic intentions, self-concepts and school engagement are needed for young people to achieve. These agency dimensions are important for both socio-economically advantaged and disadvantaged students, and for males and females. The findings also provide some support for the compensatory effect hypothesis, suggesting that high levels of school engagement (an indicator of self-regulation) are especially important for those from less privileged family background to enrol in higher education, in particular for females.

The results highlight the significant role of multiple structural forces shaping the development of domain-specific agency, and point to the importance of school-based interventions as a crucial counterbalance. Interventions should be designed to encourage students to aim high, to belief in themselves and providing an engaging learning environment. Moreover, there is a need for relevant guidance and information about the enrolment process and the demands of higher education.
